# Precision targeting: The dawn of artificially customized disease resistance

**DOI:** 10.1371/journal.ppat.1013942

**Published:** 2026-02-10

**Authors:** Xinyue Fan, Shanwu Lyu, Wenqian Fan, Jie Shu, Xiaofei Cheng

**Affiliations:** 1 College of Plant Protection, Northeast Agricultural University, Harbin, Heilongjiang, P. R. China; 2 Department of Biology, Queen’s University, Kingston, Ontario, Canada; University of Tübingen: Eberhard Karls Universitat Tubingen, GERMANY

## Abstract

Advanced plant disease management strategies are essential to sustainable agriculture and global food security. Advances in plant immunity have given rise to a variety of innovative disease control strategies, such as *NLR* gene transfer, RNA silencing technology, and CRISPR/Cas9-based gene disruption, as well as the use of immunity inducers. Recently, several novel resistance strategies, including the bioengineering of immunoreceptors, protease-triggered resistance design, and the sentinel approach, have enabled the customized development of disease resistance traits. These new approaches envisage a new paradigm of precision-targeted, artificially engineered resistance to enhance crop protection.

## Introduction

Plant diseases pose a severe threat to global food security, yet plants have evolved sophisticated defense mechanisms against pathogen invasion. Over the past century, cumulative research has elucidated key mechanisms of plant disease resistance, such as plant innate immunity, RNA silencing (RNAi), and systemic acquired resistance (SAR). These insights have informed iterative resistance strategies that integrate traditional breeding with advanced biotechnological tools to improve plant disease resistance. This review summarizes developments in plant immunology and their potential to help develop resistant crops for sustainable agriculture.

## What historical foundations shaped early plant disease control strategies?

**Fig 1:**
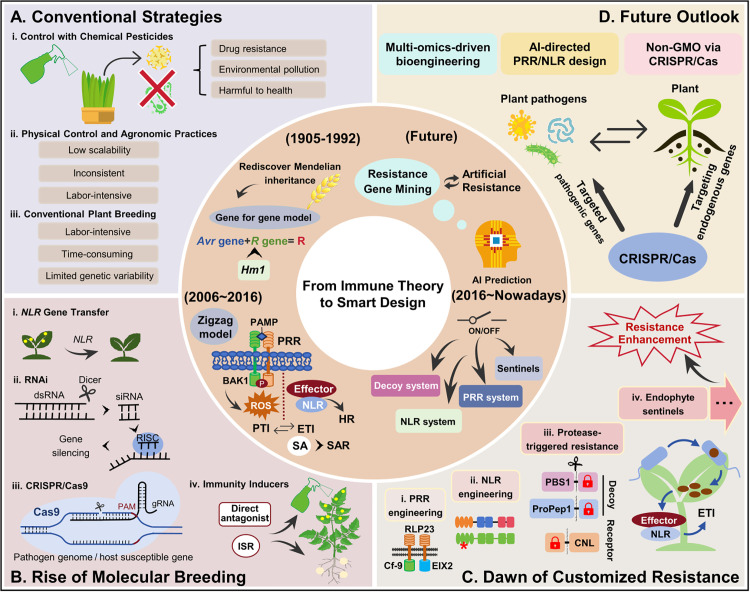
Advancement of plant disease resistance strategies toward artificially customized resistance. **(A)** Conventional strategies: **(i)** Control with chemical pesticides; **(ii)** Physical control and agronomic practices; **(iii)** Conventional plant breeding. **(B)** Rise of molecular breeding (2006-nowadays): **(i)** *NLR* gene transfer; (**ii)** RNA interference; (**iii)** CRISPR/Cas9-mediated gene disruption; (**iv)** Immunity inducers. **(C)** Dawn of customized resistance (2016-nowadays): Application of customized resistance for PRR, NLR, and protease-triggered bioengineering and endophyte sentinels. The asterisk marks the mutation site; the scissor icon indicates protease cleavage; the lock icons represent the blocking peptides. **(D)** Future outlook: Prospects for artificially customized resistance through multi-omics integration, AI-directed immnoreceptor design, and CRISPR/Cas-based non-transgenic strategies. Abbreviations: AI, artificial intelligence; *Avr*, avirulence gene; Cf-9, *Cladosporium fulvum* resistance gene 9; CNL, CC or CC_R_-NB-ARC^*^-LRR; ETI, effector-triggered immunity; EIX2, ethylene-inducing xylanase receptor 2; HR, hypersensitive response; *Hm1*, *Helminthosporium maydis 1*; ISR, induced systemic resistance; NLR, nucleotide-binding and leucine-rich-repeat immune receptor; PAM, protospacer adjacent motif; PAMP, pathogen-associated molecular pattern; PBS1, AvrPphB susceptible 1; ProPep1, precursor of plant elicitor peptide 1; PTI, PAMP-triggered immunity; PRR, pattern recognition receptor; *R* gene, resistance gene; RISC, RNA-induced silencing complex; RLP23, receptor-like protein 23; ROS, reactive oxygen species; SA, salicylic acid; SAR, systemic acquired resistance.

The period from 1905 to 1992 is known as the “early foundational phase” of plant disease resistance research. Following the rediscovery of Mendel’s laws of inheritance, the field of plant immunology began with the identification of wheat’s resistance to rust fungal pathogens. In 1942, Harold H. Flor proposed the gene-for-gene model to explain the interaction between a plant resistance gene (*R* gene) and a pathogen avirulence gene. In 1992, Johal and Briggs cloned the maize *Hm1* gene, which detoxifies the HC toxin produced by *Helminthosporium carbonum*, which validated the gene-for-gene model and accelerated the cloning of *R* genes [1]. These milestones have led to the cloning of hundreds of *R* genes to date, as well as the widespread acceptance of the gene-for-gene model. Disease control during this period predominantly relied on the application of chemical pesticides, physical controls, agronomic practices, and conventional plant breeding (Fig 1A). While these strategies have significantly contributed to the development of agriculture, they are often labor-intensive and energy-intensive, and can result in severe drug resistance, environmental pollution, and food safety issues.

## In what ways has plant molecular immunology advanced plant disease control?

Thanks to the rapid progress of molecular biology and information technology, plant immunology has undergone a short transitional period and entered the “molecular integration stage”. This period was characterized by the introduction of the zigzag model of plant innate immunity by Jonathan D.G. Jones and Jeffery L. Dangl in 2006 [2]. This model unified pattern-triggered immunity (PTI) and effector-triggered immunity (ETI), thereby establishing a foundational framework for contemporary plant innate immunity. PTI utilizes cell-surface pattern recognition receptors (PRRs, such as FLS2) to detect pathogen-associated molecular patterns and trigger broad-spectrum defense responses. In response, pathogens secrete effectors to suppress PTI and facilitate infection. However, plants have evolved intracellular nucleotide-binding leucine-rich repeat receptors (NLRs) to recognize these effectors, resulting in robust ETI, such as the hypersensitive response (HR) [2]. Since each NLR typically recognizes a limited set of specific pathogen effectors, NLRs usually confer species- or strain-specific resistance [3]. PTI and ETI interact dynamically, enhance each other mutually, and exhibit a high degree of coordination. Although the mechanism of RNAi and its significant antiviral role were discovered during the transitional period, its intrinsic connection to plant immunity through calcium signaling and the calmodulin-binding transcription factor CAMTA3 was only recently discovered [4]. Recent discoveries have further extended the role of RNAi in resisting fungi and oomycetes through bidirectional cross-kingdom silencing [4].

A comprehensive understanding of plant disease resistance mechanisms has led to the development of new disease control strategies, including *NLR* gene transfer, RNAi technology, CRISPR/Cas9-based gene disruption, as well as the utilization of immunity inducers (Fig 1B). Pioneering studies have successfully transferred a few *NLR* genes within and across species boundaries, and even between genera [5]. The stacking of multiple *NLR* genes was therefore proposed to achieve broad-spectrum resistance [6]. RNAi-based strategies, including host-induced gene silencing (HIGS), spray-induced gene silencing (SIGS), and microbe-induced gene silencing (MIGS), have been developed to deliver silencing molecules for disease control [7]. Advances in dsRNA delivery strategies, such as nanoparticle-mediated systems and optimized microbial vectors, have improved the stability and efficacy of RNAi [7]. These approaches have been successfully applied against viruses, fungi, and insects in laboratory conditions [7]. CRISPR/Cas9 and its modified variants have been used to directly target pathogen genomes or host susceptibility genes, such as *MLO* and *SWEET*, to bolster resistance [8]. Plant immunity inducers have also been developed for controlling plant diseases [9]. Key examples include beneficial microorganisms such as *Pseudomonas* spp., *Bacillus* spp., and *Azospirillum* spp., as well as oligosaccharides (OGAs), proteins, peptides, plant hormones, and their functional analogs [9].

## Why and how to bioengineer artificially customized resistance?

The idea of manipulating immune components directly to controllably activate the plant immune system has been around for decades, but it has only recently become a reality for crop breeders. In theory, this enables us to design resistance combinations that are specifically tailored to the prevalent pathogen types in a particular region. In 2016, a pioneering study replaced the AvrPphB cleavage site within the PBS1, the decoy of the NLR RPS5, with the tobacco etch virus (TEV) NIa-Pro protease cleavage site. This resulted in the activation of PBS1-RPS5-mediated immunity when challenged with TEV [10]. This decoy engineering strategy marks a paradigm shift and paves the way for the development of artificially tailored disease resistance (Fig 1C). Building on this logic, Fan et al. (2025) further extended this decoy strategy to plant elicitor peptide precursors (ProPeps). Replacing the metacaspase-4 cleavage site in the *Arabidopsis thaliana* ProPep1 with the recognition sequence of the turnip mosaic virus (TuMV)-encoded NIa-Pro or the *Pseudomonas syringae* pv. *tomato* DC3000 (DC300)-encoded AvrPphB enables the engineered ProPep1 to confer resistance against TuMV or DC3000 [11]. Unlike the aforementioned strategies, which depend on the modification of independent decoy proteins, a novel approach involving the engineering of autoactive NLRs has recently emerged. Wang et al. (2025) introduced a flexible peptide sequence carrying a pathogen-specific protease cleavage site into the N-terminal coiled-coil domain of autoactive CNLs [12]. When pathogens invade host plants, the engineered NLRs are cleaved by pathogen-derived proteases, which in turn release the autoactive domain to initiate ETI. This engineered system has successfully conferred artificial resistance against potyviruses in both *Nicotiana benthamiana* and soybean. A key advantage of this strategy is that the NLRs themselves serve as the pathogen-sensing components, thus eliminating the need for an independent decoy protein [12].

The bioengineering of NLR and PRR, either by swapping the C-terminal intracellular domain (IC domain) of PRRs or by incorporating the natural integration of effector-target domains of NLRs, allows us to alter the specificity of these immunoreceptors to create customized resistance. For example, replacing the IC domain of RLP23 and related PRRs provides robust, yield-preserving resistance against multiple pathogens in tomato, rice, and poplar [13]. Integrating the natural fusion of effector-target domains (e.g., WRKY, HMA, BED zinc finger, kinase, and Exo70) into the C-terminal of NLRs can generate precise, broad-spectrum disease resistance without altering the core architecture of NLRs [14]. The NLR-nanobody fusion strategy involves replacing the LRR domain, which is responsible for effector recognition in NLRs, with single-domain antibodies [15]. Rational or random mutagenesis of the NLR combined with resistance screening has also been reported to evolve artificial resistance [16]. In addition, a novel sentinel strategy involving the genetic engineering of plant endophytes to express effectors recognizable by host NLR receptors upon sensing pathogen invasion has recently been proposed for broad-spectrum disease resistance [17].

## What are the prospects for artificially customized resistance?

Although it is highly appealing, artificially customized resistance currently faces several major challenges, such as insufficient research into pathogenic proteases and their recognition sites, a lack of decoy molecules, and an absence of an efficient AI-directed NLR design platform. The following are potential future research directions and trends in artificially customized resistance (Fig 1D): (1) **AI and Multi-Omics-Driven Bioengineering**: AI-driven multi-omics (genomics, transcriptomics, and proteomics), structural prediction, and cross-species screening can discover novel domains for PRR and NLR bioengineering, and pathogen proteases and their cleavage motifs for decoy engineering. (2) **AI-directed PRR and NLR Design:** As AI-driven protein design technology continues to advance, e.g., ProteinMPNN and proteinBASE, the direct design of PRRs and NLRs with high efficiency and low off-target effects based on pathogen effectors or PAMPs will become a reality. (3) **Mechanism-directed Improvement**: Advances in plant immunity will certainly accelerate the development of tunable and deployable artificial resistance systems while minimizing fitness trade-offs. For instance, recent research has found that co-transferring sensor and helper NLRs can overcome the restricted taxonomic functionality [18]. (4) **Non-Transgenic Solutions**: Due to public concern about GMOs, CRISPR/Cas-mediated base editing and prime editing enable the direct modification of endogenous decoys to produce non-GMO resistant varieties. In addition to these technical advancements, suitable regulatory frameworks and biosafety considerations must be in place for the global deployment of synthetic resistance designs in agriculture.

In conclusion, customized disease resistance strategies represent a paradigm shift from gene-for-gene models to mechanism-based engineering for sustainable agriculture. By harnessing interdisciplinary tools such as AI, structural biology, and genome editing, these strategies are poised to cultivate crops with enhanced stress tolerance and broader disease resistance, thereby ensuring food security and environmental sustainability.
